# Transitional safety incidents as reported by patients and healthcare professionals in the Netherlands: A descriptive study

**DOI:** 10.1080/13814788.2018.1543396

**Published:** 2019-03-29

**Authors:** Judith M. Poldervaart, Marije A. van Melle, Leida J. Reijnders, Niek J. de Wit, Dorien L. Zwart

**Affiliations:** a Julius Center for Health Sciences and Primary Care, University Medical Center Utrecht, Utrecht, The Netherlands;; b Institute for Training of General Practitioners Utrecht, Zeist, The Netherlands

**Keywords:** Continuity of care, transitional care, primary care, medical record, transitional safety incidents

## Abstract

**Background:** Care transitions between general practice and hospital are hazardous regarding patient safety. For developing an improvement strategy adjusted to local settings, understanding of type and potential causes of transitional safety incidents (TSIs) is needed.

**Objectives:** To provide a broad overview of the nature of TSIs reported by patients and healthcare professionals.

**Methods:** We collected data (2011–2015) from three hospitals and 56 affiliated general practitioners (GPs) in two Dutch regions (one urban, one rural). We collected data from patients through a survey, interviews and incident reporting weeks, and from GPs and hospital specialists through incident reporting systems, surveys, interviews and focus group discussions. We classified reported TSIs according to type, cause and severity.

**Results:** In total, 548 TSIs were reported by 411 patients and 137 healthcare professionals; 368 of 548 TSI reports contained sufficient information for classification into aspects of the care transition process, 191 of 548 for cause, and 149 of 548 for severity. Most TSIs concerned handover correspondence from hospital to GP (26%), referral (14%) and communication/collaboration (14%). Concerning cause, reported TSIs could be attributed to organizational (48%) and human factors (43%). Twenty-four percent concerned unsafe situations, 45% near misses and 31% adverse events. Patients and healthcare professionals reported differently on referral (17% vs 9%), repeated diagnostic testing (20% vs 1%), and uncertainty about assigned responsible physician (10% vs 3%).

**Conclusion:** Reported TSIs typically concerned informational discontinuity. One third caused harm to the patient. Patients report different TSIs than healthcare professionals, suggesting a different view.

KEYMESSAGES

- Most transitional safety incident reports concern informational discontinuity, such as missing handover correspondence from hospital to general practitioner or inadequate communication or collaboration.
- One-third of the reports on transitional safety incidents were harmful for the patient.
- Patients report on different transitional safety incidents than healthcare professionals, suggesting a different view on transitional patient safety.

## Introduction

During the numerous transitions in healthcare between general practitioners (GPs) and hospital, patients are known to be at increased risk for safety incidents because of several hazards across the interface [1–4]. Examples are inadequate transfer of medical information to the next care level, such as diagnoses, test results, or changes in medication [5]. Internationally, research has shown that many transitional safety incidents (TSIs) were linked to miscommunication during the discharge process [6]. Furthermore, after referral from the GP to the hospital, information needs to be up-to-date and complete to guarantee a safe transition. However, hospital specialists consider only a minority of referral letters to be adequate [7]. Moreover, the electronic medical records of primary and hospital care are not linked, which impedes direct electronic information transfer between settings.

In the Netherlands, little is known either about number or nature of TSIs. While knowledge on exact incidence may spur a sense of urgency for change, understanding ‘what goes wrong’ is vital too. Indeed, profound knowledge about the content of incidents will guide future solutions and help to prioritize action. In addition, feedback to stakeholders on both parts will create a sense of urgency necessary for improvement [8].

The current study is conducted within the context of the Transitional Incident Prevention Programme (TIPP) study, which aimed to evaluate a multifaceted intervention programme to prevent TSIs in the Netherlands [9]. We aimed to explore type, causes and severity of TSIs as reported by Dutch patients or healthcare professionals and describe differences between patients and professionals.

## Methods

We purposefully collected TSIs through different methods, as safety incident reports differ depending on source as well as reporting method [10, 11]. Using both qualitative and quantitative methods, we collected reports on TSIs from healthcare professionals in primary and hospital care, as well as from patients.

According to literature, we defined a TSI as: ‘any unintended or unexpected event in patient care between different healthcare organizations (in our case between GP and hospital) which could have led or did lead to harm to a patient’ [12]. Furthermore, we defined a transition as ‘all movements of patients or patient information between GP and hospital (that is, referral from the GP to the outpatient clinic or medical department of the hospital, discharge from the outpatient clinic or medical department of the hospital back to the GP, and simultaneous treatment by both the hospital specialist and the GP.’

### Ethics

According to Dutch law, the TIPP study was exempted from formal medical ethical approval by the medical ethical committee University Medical Center Utrecht, The Netherlands (METC decision 13/142).

### Setting and study population

Between 2011 and 2015 we collected data on TSIs from three hospitals and 56 affiliated general practices from two regions in the Netherlands. In the urbanized area of Utrecht, data was collected in the departments of cardiology and gastroenterology of two hospitals, the University Medical Center Utrecht and the Diakonessenhuis Utrecht. In the rural region of Hardenberg, data was collected in the departments of cardiology, gastroenterology and internal medicine of the Röpcke-Zweers Hospital.

### Data collection

See Table 1 for all different data sources. Current overview applied an observational approach. We collected retrospective data from the incident reporting systems in the three hospitals. In addition, we prospectively collected patient data, using the Transitional Risk and Incident Questionnaire (TRIQ), and through interviews. For healthcare professionals, we prospectively collected data through a case report form and through focus group discussions. Finally, we collected data on TSIs reported by patients or healthcare professionals in the participating hospitals and general practices during so-called ‘incident reporting weeks.’ Exclusion from analysis occurred when the incident was not related to a transition between GP and hospital. We now provide detailed information on data collection per data source.

**Table 1.** Flowchart of data sources and number of reported TSIs by patients and healthcare professionals.

	Period of data collection	Number of participants	Number of reported TSIs^a^
Total number of participants		703	548
Patients		470	411
Survey (TRIQ)	2014–2015	239^b^	399
Interview	2013–2014	13^c^	9^d^
Reporting weeks	2014–2015	4	3
Healthcare professionals		233	137
Incident reporting systems	2011–2014	44	30
Case reports	2014	74^e^	69
Focus groups	2014	98^f^	21
Reporting weeks	2014–2015	17	17

TSI: transitional safety incident; TRIQ: Transitional Risk and Incident Questionnaire.

a One participant could report on more than one incident. This is the number of reported TSIs after exclusion when the incident was not deemed transitional.

b 239 of 454 patients completed the TRIQ questionnaire; response = 53%.

c 19 patients signed up for an interview; non-response in six patients, reasons: deemed too sick; language barrier; no incident; living too far away; not able to reach patient. Patients’ age ranged from 6 (of whom the parents were interviewed) to 82 years, the median age was 60 years.

d One patient reported two TSIs.

e 300 healthcare professionals were approached; response = 25%.

f 98 healthcare professionals participated in 12 focus groups: seven groups with GPs, five with hospital specialists.

### Incident reporting systems

We retrospectively collected safety incidents that were reported through the existing internal ‘incident reporting systems’ of the participating hospitals and general practices between 2011 and 2014 and assessed whether these concerned TSIs or not.

### Patient survey

Between November 2014 and January 2015, patients visiting the outpatient clinics of participating hospital departments were asked to digitally (through internet questionnaire) fill in the TRIQ questionnaire, which is a validated questionnaire developed to measure experienced TSIs as well as perceptions of patients on transitional patient safety [13]. For the current overview, we only used the data collected with the TRIQ questions whether patients experienced a TSI (dimension C ‘information exchange’ (items C1, C2, C4, C6–C9) and dimension D ‘collaboration between GP and hospital physician’ (items D1–D8). We asked patients to explain the background and details of the event. The research team subsequently assessed these reported events, and classified them as TSI or not.

### Patient interviews

We used a convenience sampling strategy to recruit participants that either had experienced a TSI or reported to have been at risk. Semi-structured interviews were conducted between October 2013 and June 2014. Patients were approached for the interview by their treating physician. After patients consented, they were approached by phone by a nurse to receive additional information and given an interview date. The interviews were conducted by one of three researchers (IM, LR, MM), took 36–104 min, and were taped, typed out verbatim and subsequently analysed by two researchers (IM, LR). The interviews focused on patients’ experiences during their transitional patient journey that preceded the TSI. These interviews were originally used to assess concordance with medical records, as published before [12].

### Case reports by GPs and hospital care professionals

In April 2014, GPs and hospital care professionals from both regions were asked via email to report one recently experienced TSI and provide additional information on the circumstances preceding the TSI, using a digital anonymous incident reporting system.

### Focus group discussions of GPs and hospital specialists

We asked all GPs and hospital specialists in our two regions to participate in focus group discussions. In April and May 2014, focus group discussions were held with GPs and hospital specialists, which were taped and typed out verbatim. Key themes were individual experiences and perceptions of transitional patient safety, perceived cultural differences between primary and secondary care regarding the safety climate as well as suggestions for improvement. For the current study, we extracted the examples of TSIs brought up by participants during these discussions.

### Incident reporting weeks

During two separate weeks in November 2014 and March 2015, patients and healthcare professionals were asked to report any TSIs they encountered, prospectively. Patients could report incidents on paper forms, which were available in waiting rooms of the participating general practices and outpatient clinics of hospitals. Healthcare professionals were asked to report incidents through their existing (hospital) or specially designed (GPs) electronic incident reporting system.

### Classification of reported TSIs

Two researchers (MM, VS) independently classified all reported TSIs according to aspects of the transitional process in which the TSI occurred, cause and severity. In case of disagreement, the incident was discussed with three members of the research team (VS, MM, DZ) until consensus was reached.

We classified TSIs according to aspects of care transition process in an existing safety taxonomy of the World Health Organization adjusted to transitional patient safety (Supplementary Table S1^Table 1.^) [14].

If sufficient information was available, causes of reported TSIs were determined and systematically classified using the Eindhoven Classification Model (ECM) (Supplementary Table S2) [15]. This method classifies root causes in five separate categories: technical, organizational, human, patient-related, and others.

**Figure 1.**
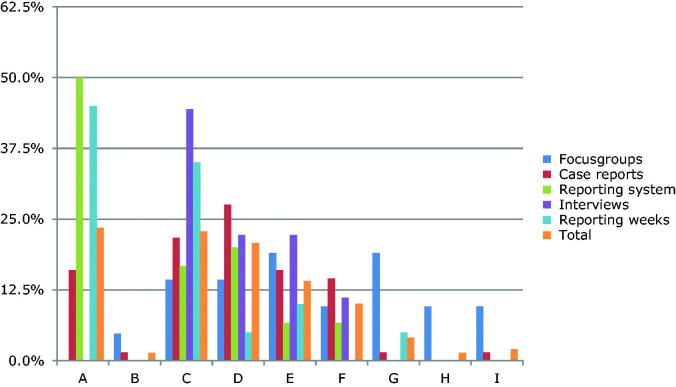
Classification by severity of transitional safety incidents according to NCC-MERP index. Category A: unsafe situation (no error); these concern events that have the capacity to cause error. Category B: near miss (error, no harm); an error occurred but did not reach the patient. Category C: near miss (error, no harm); an error occurred that reached the patient but no harm was caused. Category D: near miss (error, no harm); an error occurred, reached the patient and required monitoring/intervention to preclude harm. Category E: adverse event (error and harm); error that may have contributed/resulted in temporary harm and required intervention. Category F: adverse event (error and harm); error that may have contributed to or resulted in temporary harm and required initial or prolonged hospitalization. Category G: adverse event (error and harm); error that may have contributed to or resulted in permanent harm. Category H: adverse event (error and harm); error that required intervention necessary to sustain life. Category I: adverse event (error and death); error that may have contributed to or resulted in patient’s death.

To classify the severity of harm, we used the categories of the National Coordinating Council for Medication Error Reporting and Prevention (NCC-MERP) index (Figure 1) [16]. This index distinguishes four main categories of severity, from an unsafe situation and near miss to adverse event.

### Data analysis

The TSIs were extracted from the different data sources and listed. For each TSI we then determined type, causes and severity of harm, but only if enough information on the TSI was available necessary for classification. In case of doubt, we excluded the TSI from classification. Subsequently, we performed descriptive analyses for type, cause and severity per data source in SPSS. Concerning the qualitative data from the focus groups, we extracted and analysed the reported TSIs similar to other data sources.

## Results

In total, 548 TSI reports were collected: 470 patients reported 411 incidents, and 233 healthcare professionals reported 137 incidents. See Table 1 for numbers of TSIs per data source.

### Classification of TSIs

For 368 out of 548 (67%) reported TSIs sufficient information was available for classification according to aspects of the care transition process. In particular, data from the patient survey (TRIQ) often missed sufficient information: 180 of 399 (45%) TSIs reported through TRIQ contained insufficient information for classification. The 368 TSI reports often could be classified to more than one aspect of the care transition process, resulting in 672 classifications. In total, 171 of 672 TSIs (26%, range 22–50% within different data sources) were attributed to a problem in either handover of information from hospital to GP, or vice versa in 97 of 672 TSIs (14%, range 0–19%), or failing communication/collaboration between healthcare professionals in 93 of 672 TSIs (14%, range 0–22%) (Table 2).

**Table 2.** Classification of 368 of 548 TSIs into aspects of the care transition process, stratified per data source.

	Total		TRIQ		Interviews		Reporting weeks		Focus groups		Case reports		Reporting system	
Total number of classifications^a^	*n* = 672		*n* = 420		*n* = 22		*n* = 37		*n* = 45		*n* = 110		*n* = 38	
1. Handover correspondence to GP	172	25.6%	99	23.6%	6	27.3%	12	32.4%	12	26.7%	24	21.8%	19	50.0%
2. Referral correspondence from GP to hospital	97	14.4%	78	18.6%	0	0%	2	5.4%	5	11.1%	12	10.9%	0	0%
3. Communication/collaboration^c^	93	13.8%	57	13.6%	0	0%	8	21.6%	6	13.3%	17	15.5%	5	13.2%
4. Diagnostic testing	91	13.5%	87	20.7%	1	4.5%	0	0%	1	2.2%	2	1.8%	0	0%
5. Medication prescription	72	10.7%	33	7.9%	2	9.1%	4	10.8%	7	15.6%	22	20.0%	4	10.5%
6. Assignment of responsible physician	52	7.7%	45	10.7%	1	4.5%	1	2.7%	1	2.2%	3	2.7%	1	2.6%
7. Discharge process^b^	23	3.4%	7	1.7%	2	9.1%	2	5.4%	1	2.2%	7	6.4%	4	10.5%
8. Diagnostic reasoning	22	3.3%	2	0.5%	3	13.6%	3	8.1%	7	15.6%	6	5.5%	1	2.6%
9. Accessibility of care	14	2.1%	8	1.9%	1	4.5%	2	5.4%	0	0%	2	1.8%	1	2.6%
10. Involvement of multiple hospitals	10	1.5%	2	0.5%	2	9.1%	1	2.7%	1	2.2%	3	2.7%	1	2.6%
11. Triage of urgency	7	1.0%	0	0%	2	9.1%	0	0%	1	2.2%	2	1.8%	2	5.3%
12. Out-of-hours care	6	0.9%	0	0%	1	4.5%	0	0%	0	0%	5	4.5%	0	0%
13. Involvement of multiple specialties	6	0.9%	0	0%	1	4.5%	1	2.7%	1	2.2%	3	2.7%	0	0%
14. Internal referral	4	0.6%	1	0.2%	0	0%	1	2.7%	0	0%	2	1.8%	0	0%
15. Registration (administration)	2	0.3%	1	0.2%	0	0%	0	0%	1	2.2%	0	0%	0	0%
16. Self-care advice after discharge	1	0.1%	0	0%	0	0%	0	0%	1	2.2%	0	0%	0	0%

TRIQ: Transitional Risk and Incident Questionnaire; GP: general practitioner; TSI: transitional safety incident.

a One incident could be classified into more than one aspects of the care transition process, for example, handover correspondence to the GP and medication prescription.

b Other than correspondence.

c Besides written communication.

Healthcare professionals (*n* = 223) reported flaws in ‘handover of correspondence to GP’ as the most frequent background of a TSI (30%), followed by flaws in ‘communication/collaboration’ (16%) and in ‘medication prescription’ (16%) (Table 3). Patients’ reported TSIs (*n* = 449) concerned incidents in ‘handover of correspondence to GP’ (24%) followed by ‘repeated diagnostic testing’ (20%) and ‘referral’ (17%).

**Table 3.** Classification of 368 of 548 TSIs into aspects of care transition process, stratified for patients and healthcare providers.

	By patients		By healthcare professionals	
Aspects of the care transition process^a^	*n* = 449^a^		*n* = 223	
1. Handover correspondence to GP	106	23.6%	66	29.6%
2. Referral correspondence from GP to hospital	78	17.4%	19	8.5%
3. Communication/collaboration	58	12.9%	35	15.7%
4. Diagnostic testing	88	19.6%	3	1.3%
5. Medication prescription	37	8.2%	35	15.7%
6. Assignment of responsible physician	46	10.2%	6	2.7%
7. Discharge process^a^	10	2.2%	13	5.8%
8. Diagnostic reasoning	6	1.3%	16	7.2%
9. Accessibility of care	9	2.0%	5	2.2%
10. Involvement of multiple hospitals	5	1.1%	5	2.2%
11. Triage of urgency	2	0.4%	5	2.2%
12. Out-of-hours care	1	0.2%	5	2.2%
13. Involvement of multiple specialties	1	0.2%	5	2.2%
14. Internal referral	1	0.2%	3	1.3%
15. Registration (administration)	1	0.2%	1	0.4%
16. Self-care advice after discharge	0	0%	1	0.4%

GP: general practitioner; TSI: transitional safety incident

a A TSI could be classified into more than one aspects of the care transition process.

b Other than written correspondence

Compared to healthcare professionals, patients more often reported ‘omission of referral information’ (17% vs 9%) and ‘repeated diagnostic testing’ (20% vs 1%) (Table 3). Alternatively, healthcare professionals reported TSIs that more often concerned problems with medication (16% vs 8%).

In total, 191 of the 548 (35%) TSI reports contained sufficient information for identifying at least one cause, using the ECM criteria (Table 4). ‘Organizational factors’ (48%) and ‘human factors’ (43%) were the two most frequently identified categories for causes.

**Table 4.** Classification of 191 of 548 TSIs by cause according to the Eindhoven Classification Model (ECM), stratified for the different data sources.

	Total		TRIQ		Interviews		Reporting weeks		Focus groups		Case reports		Reporting system	
Causes	*n* = 493		*n* = 299		*n* = 34		*n* = 88		*n* = 37		*n* = 11		*n* = 24	
Technical	4	0.8%	0	0%	0	0%	1	1.1%	2	5.4%	0	0.0%	1	4.2%
Organizational	104	21.1%	11	3.7%	14	41.2%	40	45.5%	22	59.5%	5	45.5%	12	50.0%
Human	93	18.9%	6	2.0%	17	50.0%	41	46.6%	12	32.4%	6	54.5%	11	45.8%
Patient-related	9	1.8%	4	1.3%	2	3.1%	2	2.3%	1	1.4%	0	0%	0	0%
Unclassifiable	283	57.4%	278	93.0%	1	1.5%	4	4.5%	0	0%	0	0%	0	0%

TRIQ: Transitional Risk and Incident Questionnaire.

Of the 548 TSIs, 149 (27%) could be classified according to the NCC-MERP (Figure 1). In total, 35 of 149 (24%) TSI reports were classified as unsafe situations (category A), 45% as near misses (category B to D) and 31% as adverse events (categories E to I).

Different data sources revealed different severity. The focus group discussions revealed the most harmful incidents with two thirds being adverse events, as opposed to the incident reporting weeks and the reporting systems, in which half were classified as unsafe situations.

Three of 149 incidents (2%) had fatal consequences (category I), of which two were from focus group discussions and one was a case report. One concerned a hip fracture not recognized during the diagnostic process and one concerned a carcinoma diagnosed after failure of follow-up of a suspicious test result. Both regarded vulnerable patients (elderly or cognitively impaired). The third concerned a patient with a newly diagnosed abdominal aneurysm, of whom the hospital had provided no handover correspondence to the GP and when the patient presented with symptoms, those were not linked by the GP to an aneurysm and the patient died.

## Discussion

### Main findings

We identified a total of 548 TSIs reported by patients or healthcare professionals through seven different data sources. Correspondence and communication between hospital and GP, during referral, discharge, and simultaneous care at the outpatient hospital care were the most vulnerable aspects of the care transition process, and TSIs were mainly caused by organizational factors.

Furthermore, whereas healthcare professionals noted more medication discrepancies, patients noted redundant testing and uncertainty about the first responsible physician more frequently. Although most reported TSIs did not cause patient harm, one-third did, of which a minority with fatal consequences. Many of the reported TSIs provide opportunities for prevention of adverse health outcomes.

### Strengths and limitations

Our large sample of TSIs with its rich and heterogeneous spectrum using all the different data sources, each with their own logistics and methodology, engendered some limitations. Concerning generalizability, undoubtedly the presented overview is an underrepresentation of the actual occurrence of TSIs, as underreporting of safety issues is common. For instance, patients are unlikely to report unsafe situations that they do not notice and healthcare professionals are known to underreport minor incidents [17]. In addition, patients tend to be quite forgiving to their healthcare professionals because of their dependent relationship [13, 18]. Although possibly not all types of TSIs are equally represented in our study, we believe our results provide a valid and moreover broad overview of TSIs as occurring in clinical practice and can guide future steps towards improvement. Of course, we cannot draw any conclusions on the incidence of subtypes of TSIs, as our methods were not suited for judgment on frequencies.

Additionally, often we could not classify the TSIs because of insufficient details reported. This mainly concerned patient-reported TSIs from the TRIQ survey, which should be interpreted with caution.

Notwithstanding these limitations concerning our choice of data sampling, we believe this approach was worthwhile since we optimally used information that sometimes was already available, as well as assuring this broader view as explained above.

### Interpretation of study results

Inadequate information exchange between general practice and hospital was most frequently mentioned as a cause of TSIs. This is in line with the literature: a survey among 4720 physicians demonstrated that only 35% of hospital specialists always received referral information, and only 62% of GPs received handover correspondence [19]. Kripalani et al. reported that in the US 51–77% of the discharge summaries were not received by GPs within four weeks [5].

Interestingly, in our study, 20% of patients reported that diagnostic testing was repeated in the hospital while only 1% of healthcare professionals mentioned this. Although possibly difficult for patients to assess whether repeated testing is truly redundant, the patient is the only one present in all phases of the healthcare process. Therefore, patients might be able to reveal issues that healthcare professionals are simply unaware of [10, 20]. Little is known on repeated diagnostic testing. One study demonstrated that a 50% reduction in redundant laboratory testing was possible with better sharing of information [21]. Scrutinizing patients’ reports on redundant testing may be another fruitful source to uncover unnecessary diagnostics threatening transitional safety.

As to the causes of TSIs, we mainly identified organizational and human factors. This is in contrast to a large Dutch medical record review study, which found 43% of all in-hospital incidents were patient related, followed by 32% due to human factors [22]. Smits et al. found human factors (61%) were the main cause in adverse events in the hospitalized patients, followed by 39% by patient-related factors [23]. The discrepancy might be caused by the different processes involved in transitional safety as compared to in-hospital patient safety. In transitional patient safety, well-organized and documented communication within the medical record between settings is vital. Current, frequently occurring flaws can, therefore, be seen as organizational flaws, although no definite causal effect can be concluded from the current analysis. The low percentage of patient-related causal factors is probably explained by the fact that the patient often has a fairly inactive and inexplicit role in transitional patient safety [24, 25].

Our distribution of reported TSIs was in line with the literature. Forster et al. found adverse events in 19% of the patients after discharge [26]. The TSIs reported in our focus group discussions were more severe than those identified through other data sources. A focus group setting is likely to provoke discussion on events that have had a major impact on the physician. These are inherently those with severe patient harm. The same is true for the case reports. Lastly, we identified many unsafe situations that can cause harm; however, literature on these minor TSIs is scarce. Tam et al. demonstrated unsafe situations in 10–67% of patients related to incomplete medication histories on hospital admission [27]. Although these unsafe situations may seem harmless and futile, they require time and effort of healthcare professionals to be corrected and may escalate to patient harm in the presence of other risk factors [28].

### Interpretation for clinical practice

This broad overview of different types of TSIs can be used as feedback information to assess current transitional patient safety. Results suggest TSIs are often occurring, implying urgency to improve. Our results also indicate improvement strategies should focus on exchange of information between hospitals and general practices. Other countries might benefit from our study, as most have healthcare systems where inpatient care and primary care are separate worlds [29].

## Conclusion

TSIs typically occur in the processes of information continuity such as discharge from hospital to general practice, referral vice versa or other communication. One-third of TSIs cause harm to the patient. Patients report different TSIs than healthcare professionals, suggesting a different view on transitional patient safety.

## Supplementary Material

Table S2

Table S1
