# Supplementary material for: Transitional safety incidents as reported by patients and healthcare professionals in the Netherlands: A descriptive study
Source: Eur J Gen Pract. 2019 Mar 29;25(2):77–84. doi: 10.1080/13814788.2018.1543396 (PMC6493279; doi:10.1080/13814788.2018.1543396)
Supplement: Table S2 [file IGEN_A_1543396_SM2984.docx]

**Table S2. Classification by causes of reported TSIs: the Eindhoven Classification Model (ECM): Medical Version**

| **1. Technical** |  |
| --- | --- |
| External | Any technical failures beyond the control and responsibility of the investigating organisation |
| Design | Failures due to poor design |
| Construction | Correct design which was not followed accurately during construction aspects |
| **2. Organisational** |  |
| External | Any failures at an organisational level beyond the control and responsibility of the investigating organisation |
| Transfer of Knowledge | Failures resulting from inadequate measures taken to ensure that situational or domain-specific knowledge or information is transferred to all new or inexperienced staff |
| Protocols | Failures related to the quality and availability of the protocols within the department (too complicated, inaccurate, unrealistic, absent, poorly presented) |
| Management priorities | Failures resulting from management decisions in which safety is delegated to an inferior position when faced with conflicting demands or objectives |
| Culture | Failures resulting from a collective approach and its attendant modes of behavior to risks in the investigating organisation |
| **3. Human** |  |
| External | Human failures originating beyond the control and responsibility of the investigating organisation |
| Knowledge | Inability of an individual to apply their existing knowledge to manage novel situations |
| Qualifications | Incorrect fit between an individual's qualifications, training or education and a task |
| Coordination | Lack of task coordination within the organisation or team |
| Verification | Failures in the correct and complete assessment of a situation including relevant conditions of the patient and materials to be used before starting the intervention |
| Intervention | Failures that result from faulty task planning and execution |
| Monitoring | Failures during monitoring of process or patient status during or post-intervention |
| Slips | Failures in the performance of fine motor skills |
| Tripping | Failures in whole body movements |
| **4. Patient-related** |  |
| Patient-related factor | Failures related to patient characteristics which are beyond the control of staff and influence treatment |
| **5. Unclassifiable** |  |
| Unclassifiable | For failures that cannot be classified in any other category |

TSI: transitional safety incidents
