# Supplementary material for: Transitional safety incidents as reported by patients and healthcare professionals in the Netherlands: A descriptive study
Source: Eur J Gen Pract. 2019 Mar 29;25(2):77–84. doi: 10.1080/13814788.2018.1543396 (PMC6493279; doi:10.1080/13814788.2018.1543396)
Supplement: Table S1 [file IGEN_A_1543396_SM2958.docx]

**Table S1. Classification according to aspects of the care transition process in which the transitional safety incident reports occurred**

| Aspects of the care transition process | Examples of collected transitional safety incidents |
| --- | --- |
| **1. Handover correspondence from the hospital to the GP**  Information after outpatient clinic visits or discharge letters can be delayed*, incomplete or absent. | *A patient with (normally perfectly regulated) diabetes visits the GP with repeated high glucose levels. A change in medication by the GP does not control the blood glucose levels. After 4 weeks, the GP receives a letter from the pulmonologist; the patient started with prednisone over a month ago because of his exacerbated chronic obstructive pulmonary disease.* |
| **2. Referral correspondence from the GP to the hospital**  Information from the GP to the hospital can be delayed*, incomplete or absent. | *A patient is referred to the emergency department for complaints of chest pain. However, the referral letter lacks information on medical history, although the patient tells the physician at the emergency department he has a cardiac disease, although he does not know what specifically.* |
| **3. Communication/collaboration**  This concerns all communication or signs of collaboration apart from written communication | *A GP receives a euthanasia request by telephone from a patient’s family. The patient was diagnosed with terminal cancer in hospital of which the GP was not yet informed. He recently referred the patient to hospital because of a back ache.* |
| **4. Diagnostic testing**  Diagnostic testing can be omitted, or may be performed twice redundantly. | *A GP measures lipid levels of a patient. In the hospital, the lipid levels are measured again in the same week by the internal medicine hospital care provider.* |
| **5. Medication prescription**  Incorrect or absent communication between healthcare providers on type, dosage, frequency. | *The GP prescribes bumetanide to a patient with heart failure and complaints of fluid retention. However, the patient already used furosemide which was prescribed by the cardiologist, but not communicated to the GP. The patient uses both diuretics and then visits the heart failure nurse because of complaints of dehydration, who discovers the double medication.* |
| **6. Assignment of responsible physician**  It can be unclear who is the first responsible physician for managing patient’s current disease episode; or no responsible physician is assigned at all. | *A patient with kidney failure is treated in the hospital. The GP does not know whether the hospital care provider also performs the diabetes controls for this patient.* |
| **7. Discharge process from the hospital to the GP (other than correspondence)** | *A patient is discharged from the outpatient clinic of the cardiologist but this is not communicated to the GP. The patient then does not receive controls for his cardiovascular risk management for the next 5 years.* |
| **8. Diagnostic reasoning**  The GP does not (timely) recognize complaints, and referral is delayed or misdirected. | *Patient was referred to the emergency department by the GP with suspicion of cerebrovascular accident. However, it turned out the patient had a simple urinary tract infection and was no emergency case at all, and the GP had not visited the patient first.* |
| **9. Accessibility of care** | *A patient is referred to a tertiary care hospital, but is refused as the nature of complaints is considered not complex.* |
| **10. Involvement of multiple hospitals** | *Patient is being referred by GP for complaints of back pain with radiation to the neurologist. However, after multiple tests it turned out the patient was already known with these complaints in another hospital, and wanted a second opinion.* |
| **11. Triage of urgency** | *A patient was referred to the ophthalmologist (OP) with complaints of vision loss and eyelid swelling. The hospital secretary booked a consultation with an optometrist instead, who diagnosed tear film insufficiency. When the patient returned after several weeks to the GP with persistent complaints, the GP referred the patient to the OP in a different hospital. Here, the OP diagnosed an orbital meningioma for which the patient had neurosurgical treatment.* |
| **12. Out-of-hours care**  This concerns care between 17:00h and 8:00h and during the weekends | *During an out-patient clinic visit, a patient is coincidentally diagnosed with an abdominal aneurysm, which is not communicated to the GP. After an emergency visit to the GP with a backache, the patient dies at the GP out-of-hours service, because the GP does not recognize the ruptured aneurysm in time.* |
| **13.Involvement of multiple medical specialties** | *Both the cardiologist and internal medicine hospital care provider treat the patient, but because they do not report to each other, they are not aware of each other and both perform a cardiac stress test. The patient complaints about this to the GP.* |
| **14. Internal referral** | *A patient is known at the GP with a thyroid disorder, for which she is adequately treated by the GP for several years now. Because of an acute problem, she was referred to the ear-nose-throat-(ENT) specialist. The ENT-specialist internally refers her to the internal medicine for her (stable) thyroid problem.* |
| **15. Registration (administration)** | *A patient goes into cardiac arrest during an endoscopic procedure and is successfully resuscitated. However, afterwards it turned out the patient had requested a do-not-resuscitate policy, which was not communicated by the GP.* |
| **16. Self-care advice after discharge**  No or inadequate communication to patient to manage condition at home | *The patient did not receive adequate advice when at home, and makes an appointment with the GP to answer all his questions after discharge.* |

GP: general practitioner

* delay communication was defined as 14 days in our study
